# Hepatic Zonation in MASLD: Old Question, New Challenge in the Era of Spatial Omics

**DOI:** 10.3390/ijms262110701

**Published:** 2025-11-03

**Authors:** Erika Paolini, Miriam Longo, Marica Meroni, Paola Dongiovanni

**Affiliations:** Medicine and Metabolic Diseases, Fondazione IRCCS Cà Granda, Ospedale Maggiore Policlinico, 20122 Milan, Italy; eryka.paolini.93@gmail.com (E.P.); longo.miriam92@gmail.com (M.L.)

**Keywords:** MASLD, spatial omics, liver zonation, precision medicine

## Abstract

Hepatic zonation reflects the concept that hepatocytes and nonparenchymal cells (NPCs) perform distinct metabolic functions, depending on their spatial localization along the porto-central axis. The maintenance of this fine-tuned organization is essential for liver homeostasis, and its disruption may contribute to liver diseases, including metabolic dysfunction-associated steatotic liver disease (MASLD). Fat overload perturbs zonal gene signatures, lipid handling and oxygen/metabolite gradients progressively leading to steatohepatitis (MASH), fibrosis and HCC, conditions in which the hepatic architecture is lost. Traditional approaches have provided valuable insights into zonation, although they lack the spatial resolution and mask the heterogeneity of NPCs. Thus, the premise of this review is to discuss how spatial omics can redefine our understanding of hepatic zonation by integrating tissue mapping with metabolic organization, specifically focusing on MASLD. The advent of spatial omics accelerates knowledge regarding MASLD pathophysiology, providing more informative insights into transcriptional/translational/lipidomic/metabolomics zone-specific perturbations. Emerging spatial genomics and epigenomics applications further expand this scenario, allowing for the capture of chromatin remodeling in situ. The integration of these state-of-the-art approaches, coupled with artificial intelligence (AI)-based analyses, is promising in the clinic, as it may provide novel zonal prognostic biomarkers and pave the way for precision-medicine strategies targeting zonal switching.

## 1. Introduction

Metabolic dysfunction-associated steatotic liver disease (MASLD) refers to hepatic fat infiltration accounting for more than 5% of the total liver weight plus one of five criteria: central obesity, hypertension, glucose intolerance/type 2 diabetes mellitus, hypertriglyceridemia, or reduced HDL cholesterol and, nowadays, it is recognized as the most frequent liver disease, affecting 35% of the global population [1]. MASLD ranges from the benign and asymptomatic condition of steatotic liver disease (SLD) to metabolic dysfunction-associated steatohepatitis (MASH) in 15–35% of cases [2]. The latter is featured by lobular inflammation and ballooning degeneration and can progress toward more severe conditions, such as fibrosis (40%) that in turn may lead to cirrhosis (25%) up to hepatocellular carcinoma (HCC) (2–4%), the third most common cause of cancer death [3]. MASLD is strongly linked to insulin resistance (IR) and type 2 diabetes mellitus (T2DM), leading to high circulating plasma free fatty acids (FFA) and triglycerides that are uptaken by the liver [4]. The sustained fat overload causes the hepatic injury cascade encompassing reactive oxygen species (ROS) overproduction, mitochondrial dysfunction, both mitochondrial (mtDNA) and nuclear DNA damage, endoplasmic reticulum (ER) stress and inflammation [5].

In the last decade, the health burden of MASLD has rapidly increased in the global population owing to the epidemic obesity and the insufficient clinical and diagnostic advances, coupled with patients’ unawareness until the disease reaches its more severe stages [1]. Therefore, emerging studies focused on tackling MASLD in the early stages to prevent disease progression [6]. The liver is the pivotal organ involved in physiological homeostasis and energy metabolism. Furthermore, it manages the synthesis of several circulating proteins, such as albumin and coagulation factors, as well as the primary defense strategies for drug and alcohol detoxification [7]. Proper liver function hinges on hexagonal-shaped anatomical units defined as lobules, which have a specific spatial distribution of metabolic functions among different lobular zones. Blood rich in oxygen enters the liver’s lobule at the peripheral portal tracts and drains out through the central vein. Conversely, bile flows outwards from the lobule centers and drains out through the portal bile ducts [8]. The hepatocytes (HEPs), the primary liver cell type, have a radial arrangement in cords around the sinusoidal capillaries. The latter host nonparenchymal cells (NPCs): liver sinusoidal endothelial cells (LSECs) that line the sinusoids and guarantee the metabolic flux from/to the circulation; hepatic stellate cells (HSCs) dwell in the “space of Disse” between hepatocytes and the sinusoids; hepatic macrophages named Kupffer cells (KCs) which reside within the sinusoids and protect against gut-derived bacteria; cholangiocytes that transport bile acids and waste products from hepatocytes to the intestine. Following the bloodstream gradient towards the central vein, HEPs store and release nutrients and hormones such as insulin, glucagon, growth factors, and thyroid hormones, creating a graded microenvironment. This physiological gradient spatially organizes hepatic functions along the lobule radial axis, resulting in “*liver zonation*” [9,10,11]. This compartmentalization permits different metabolic functions, even opposing ones, to occur simultaneously and allows HEPs and NPCs to perform specific tasks depending on their location.

**Figure 1:**
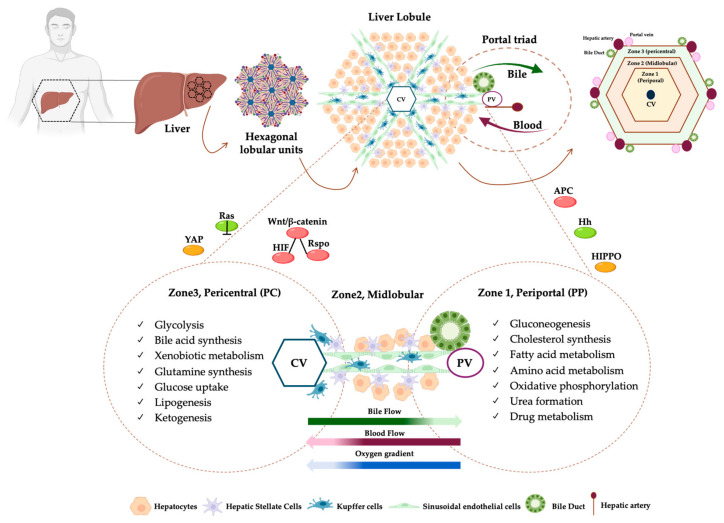
Schematic representation of the liver lobule and metabolic liver zonation. The liver is divided into different lobes (right, left, caudate and quadrate lobes), organized in honeycomb-like structures arranged in heterogeneous size and axial orientation, named lobules. The lobules depict the smallest anatomical units with a hexagonal shape with a central vein (CV) in the center and portal triads composed of a portal vein (PV), a hepatic artery (HP) and a bile duct (BD), at the periphery. Within lobules, the hepatocytes (HEPs) are interconnected as cords that radiate out from CV towards PV, thus resulting in two intertwined radial networks: the sinusoids for blood flow (red arrow) and the bile canaliculi for bile secretion (green arrow). The region surrounding the portal triads is called periportal (PP, zone 1), the one adjacent to the CV is known as pericentral (PC, zone 3), and the area in between is referred to as midlobular (zone 2) (upper panel). Hepatic parenchymal cells, namely hepatocytes, radiate out from CV towards PV. The sinusoids are surrounded by sinusoidal endothelial cells and are populated by the hepatic macrophages known as Kupffers. Moreover, the space between the endothelial cells and HEPs provides a niche for hepatic stellate cells (HSCs). Hepatocytes manage the availability of nutrients and hormones, generating a flow of bile, blood and oxygen across the PP and PC areas. In turn, this gradient contributes to specialized HEPs in distinct metabolic pathways, resulting in a phenomenon defined as *zonation.* Gluconeogenesis, cholesterol synthesis, fatty acid and amino acid metabolism, oxidative phosphorylation, urea formation and drug metabolism are preferentially located in the PP zone, which receives blood richer in oxygen and nutrients, while glycolysis, bile acid synthesis, xenobiotic metabolism, glutamine synthesis, glucose uptake, lipogenesis and ketogenesis are mainly distributed in the PC zone. Metabolic hepatic zonation may be affected by several pathways: Wnt/β-catenin-HIF-Rspo signaling is mainly located in the PC zone, whereas APC, its negative regulator, is in the PP one; Hedgehog (Hh) pathway is higher in the PP region together with Ras-dependent signaling; HIPPO pathway is mainly activated in PP following oxygen gradient whereas YAP is located in PC (lower panel).

The zonation along the periportal (PP) and perivenous or pericentral (PC) axis delineates three main areas in the hepatic acinus: zone 1 (PP zone), close to the portal triad, zone 2 (middle zone, M) and zone 3 (PC zone), close to the central vein [10,12]. A schematic representation of hepatic lobule architecture and of the different zones has been reported in Figure 1.

Gluconeogenesis, cholesterol synthesis, fatty acid and amino acid metabolism are preferentially located in the PP zone, which receives blood richer in oxygen and nutrients, while glycolysis, bile acid synthesis and xenobiotic metabolism are mainly distributed in the PC zone [9,10,13,14,15], thus underlining the pronounced heterogeneity in gene expression between hepatocytes along the PP to PC axis. In this regard, single-cell profiling of mouse hepatocytes revealed that half of the genes detected were zonated (3496 of 7277 genes) [14], and the heterogeneity among lobular zones was corroborated in another study of spatial transcriptomics in female mice livers [16]. Emerging evidence supports the notion that changes in liver zonation due to MASH correlate with impaired gene signature gradients along the PC to PP axis, thus providing novel findings to decipher the intricate MASLD pathobiology. In this review, we provided a proof of concept regarding the applicability of spatial omics to define the hepatic histological features alongside the metabolic organization. The assessment of specific metabolic changes within liver zonation during MASLD progression could strongly empower the prognostic and therapeutic strategies.

## 2. Zonation of Liver Architecture

The liver is a complex, 3D-structured and functionally heterogeneous organ that plays a pivotal role in vertebrates’ metabolic processes. It is supplied by a dual blood flow, one from the hepatic portal vein, which is enriched in nutrients, and the other from the hepatic artery that carries oxygenated blood to the liver. They converge into hepatic sinusoids and then leave the liver through the hepatic vein [17]. In humans, the liver is divided into different lobes (right, left, caudate and quadrate lobes). Each lobe consists of functionally distinct lobules that depict the smallest units with a hexagonal shape showing a central vein in the center and portal triads (portal vein, hepatic artery, and bile duct), on the boundary. In turn, the lobules are organized in honeycomb-like structures arranged in heterogeneous sizes and axial orientation [18]. Within the lobules, hepatocytes are interconnected as cords that radiate out from the PC zone towards portal triads, thus resulting in two intertwined radial networks: the sinusoids for blood flow and the bile canaliculi for bile secretion [19]. The sinusoids are surrounded by LSECs and are populated by KCs and lymphocytes. Moreover, the space between the endothelial cells and hepatocytes is known as the space of Disse, which provides a niche for HSCs, involved in extracellular matrix formation during liver damage and inflammation [20] (Figure 1).

A novel approach called spatial reconstruction exploited the positioning of HEPs on a virtual lobular axis and the inference of spatial context to investigate the mechanisms underlying spatial [14] and spatiotemporal [21] metabolic liver zonation. These studies provided specific gene expression profiles in the different lobular zones and defined positional markers. PP hepatocytes mainly express *PCK1*, *HAL*, *GLS2*, *HSD17B13*, *ASS1,* and *ARG1* whereas PC ones are zonated by *GLUL*, *CYP2E1*, *CYP1A2*, *CYP3A4,* and *OAT* [22]. Midlobular hepatocytes express *HAMP*, *HAMP2,* and *IGFBP2,* although their zonation is less defined (Table 1).

Hepatocytes pick up and discharge nutrients and hormones, thus creating a graded microenvironment of oxygen, metabolites and signaling molecules from the PP area to PC. In turn, this gradient contributes to specialize HEPs and NPCs in distinct metabolic pathways, resulting in hepatic *zonation* [23]. The division of tasks between hepatocytes and the surrounding cells is crucial to coordinate complex and even opposed processes. In addition, a temporal control of the metabolic pathways assures their alignment to fasting and feeding cycles [24]. Therefore, spatial and temporal liver zonation is essential for physiological hepatic functions since it guarantees energy homeostasis, manages metabolism of nutrients and xenobiotics, and regulates the lifecycle of proteins. The patterns of gene expression and their related enzymes change localization in response to nutrition, drugs, hormones, and other blood factors, thus emphasizing the importance of exploring the dynamic architecture and metabolic zonation to manage liver disease outcomes.

## 3. Transcriptomics Signature of Liver Zonation

Hepatic zonation may be affected by several pathways, including Wnt/β-catenin, Hedgehog (Hh) signaling, HIPPO pathway and the oxygen gradient. The Wnt/β-catenin pathway plays a key role in hepatic zonation by orchestrating distinct functions of liver metabolism and modulating the expression of ≈30% of zonated genes. β-catenin protein is the central actor in the Wnt/β-catenin molecular cascade, and its activation is crucial for metabolic patterning of the liver [25]. Its amount is regulated at the post-translational level in the absence of Wnt signals and constitutes a multicomplex with glycogen synthase kinase-3 (GSK3), adenoma polyposis coli (APC), CK1, Axin, and the protein Dishevelled (DVL). Specifically, β-catenin phosphorylation, mediated by GSK3, is necessary to recruit the ubiquitin ligase β-TRCP, which fosters its proteasomal degradation [26,27]. In the canonical pathway, Wnt ligands are secreted glycoproteins that act through an autocrine or paracrine manner by binding to a Frizzled receptor and LDL receptor–related protein 5/6 (LRP5/6) co-receptors. β-catenin, which is stored in the cytoplasm, moves into the nucleus and interplays with the TCF (T-cell factor) family to guide target gene transcription. In the homeostatic state, Wnt/β-catenin activity is mainly located in the PC area [28], whereas APC, its negative regulator, is located in the PP one. It has been demonstrated that knockout (KO) *APC* mice activated the β-catenin pathway also in the PP area [29]. In line with this phenomenon, the liver-specific *β-catenin*, *APC* and *Lrp5/6* KO mice exhibited defects in zonation patterns, thereby supporting their role in driving this process [15,29,30,31]. Moreover, it has been described that the spontaneous differentiation of liver stem cells gives rise to PP hepatocytes, and these hepatocytes switch into PC ones after Wnt/β-catenin activation [32,33]. In keeping with these results, mutations in the β-catenin gene (*CTNNB1*) leading to its aberrant activation may trigger metabolic alterations in liver cancers [34,35].

Among the Rspondin (Rspo) family proteins, which belong to the β-catenin pathway, Rspo3 is closely expressed in the liver acinus and is detected selectively in endothelial cells of the central vein. It has been demonstrated that deletion of *Rspo3* in mice disrupts the β-catenin-dependent zonation in PC [36]. Moreover, the Rspo1 receptor, LGR5, is highly expressed in damage-activated liver stem cells and exclusively in PC hepatocytes, resulting in Wnt/β-catenin signaling activation [13,37]. Consistently, its deletion in mouse liver deregulated the expression of PP and PC marker genes such as glutamine synthetase (GS) [38]. Accordingly, in hepatoma cells (HepG2), a hypoxic condition triggers LGR5 expression, supporting that the LGR5-β-catenin linkage is controlled by oxygen gradient within the liver acinus [20].

The transcriptional adaptation related to changes in oxygen levels is mainly mediated by the family of hypoxia-induced transcription factors (HIFs) [39]. Several evidence indicate that hypoxia and the Wnt/β-catenin signaling are intertwined. Indeed, HIF proteins are expressed in the PC zone, and they mediate the transcription of genes involved in hepatocytes zonation, including β-catenin [40,41,42]. In line with this data, the deletion of HIF1α in stem cells under hypoxic conditions lowered the expression of Wnt/β-catenin, alongside the tumor suppressor APC [43,44]. In particular, the PP oxygen ranges from 60–65 mm Hg (84–91 μmol/L) to about 30–35 mm Hg (42–49 μmol/L) in the PC area. Consistently, the mitochondrial content/architecture as well as the oxidative capacities are higher in PP and progressively decline toward the PC zone [45,46,47,48]. Moreover, oxygen is crucial to produce ROS, enhancing the presence of an intra-acinar redox gradient, and it has been demonstrated that perfused livers showed a redox flow from the PP to PC region [49,50]. Notably, oxygen regulates the carbohydrate metabolism in the livers of rats and cultured primary hepatocytes [51,52,53] as well as lipid metabolism, thus playing a role in the development of steatosis [54]. Indeed, glucagon, which is released as a hormone from the pancreatic α-cells towards lobules, generates a gradient drop along the PP–PC axis [32]. Accordingly, glucagon-deficient mice (*Gcg*^−/−^) showed an altered hepatic zonation that was rescued by glucagon infusion [55].

Emerging evidence proposed the Hedgehog (Hh) signaling that plays a crucial role in liver development and regeneration, as a nominee driver for hepatic zonation [56]. In adults, Hh pathways are poorly expressed in HEPs, whereas it is higher in HSCs and cholangiocytes [57,58]. Different from Wnt/β-catenin activity, Hh signaling is higher in the PP region and is involved in the regulation of zone 1 of hepatocytes. In particular, it is activated during liver damage over MASLD, cirrhosis and HCC, thus opening the spyhole in the identification of novel diagnostic and therapeutic opportunities [59,60]. Hh ligands, such as Sonic Hh, Indian Hh, and Desert Hh, bind to Ptch1/2 receptors to mitigate patched-mediated suppression of Smoothened, which activation leads to the stabilization and nuclear translocation of GLI transcription factors [61]. Two studies proposed distinct Hh signaling regulations of hepatic zonation by exploiting *Smoothened* KO mice, as follows: the first affects the Wnt/β-catenin pathway by downregulating its target genes, and the second manages the insulin-like growth factor axis through the GLI3 transcription factor [62,63]. Similarly to Hh, Ras-dependent signaling induces a PP phenotype by inhibiting PC one [64].

Finally, HIPPO signaling has been described as another modifier of hepatic zonation. When it is activated, YAP does not move into the nucleus and undergoes proteasomal degradation. Conversely, when the HIPPO pathway is turned off, YAP translocates into the nucleus and promotes the transcription of genes involved in the transdifferentiation of hepatocytes into biliary or progenitor cells. Hypoxia and HIF proteins may affect the HIPPO signaling, and in physiological conditions, it is mainly activated in PP following the oxygen gradient, whereas YAP is located in PC. In the context of liver damage, YAP is distributed across the entire lobule and the metabolic zonation is shaped to face liver repair [65]. Illustration of hepatic metabolic zonation and genes involved in this process are reported in Figure 1.

## 4. Metabolic Zonation: Physiological Status vs. MASLD

Oxygen availability guides different metabolic processes across the hepatic lobule. At the physiological level, hepatocytes that are localized in the PP area, where oxygen is more abundant and metabolic processes are energetically demanding, participate in glucose delivery, gluconeogenesis from lactate, urea synthesis, fatty acid oxidation, sulfation, and cholesterol synthesis. Conversely, PC hepatocytes are preferentially engaged in xenobiotic metabolism requiring low oxygen and are involved in glucose uptake, glycolysis from glucose, glutamine synthesis, bile acid synthesis, lipogenesis and ketogenesis [10,14,20]. Several zonated metabolic processes are interconnected between the PC–PP axis due to the recycling of substrates. During the urea cycle, PP hepatocytes detoxify ammonia to generate urea by using glutamate derived from glutamine and concurrently, PC hepatocytes exploit the surplus of glutamate to reconvert it into glutamine, thus ensuring the balance of this process [8]. Additional examples of opposite zonated tasks include PP production and PC uptake of glucose, as well as PP cholesterol biosynthesis and PC cholesterol consumption.

**Figure 2:**
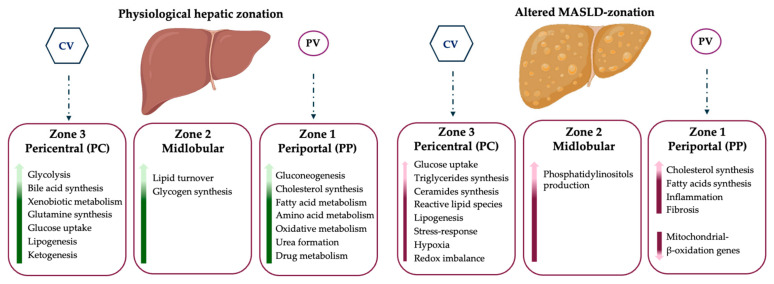
MASLD is a spatially organized disease. In a healthy liver, pericentral hepatocytes are preferentially involved in glycolysis, bile acid synthesis, xenobiotic metabolism, glutamine synthesis, glucose uptake, lipogenesis and ketogenesis, whereas periportal hepatocytes are enriched in gluconeogenesis, cholesterol synthesis, fatty acid metabolism, amino acid metabolism, oxidative metabolism, urea formation, and drug metabolism. Midlobular hepatocytes manage lipid turnover and glycogen synthesis. During MASLD, this physiological zonation becomes altered, and distinct regions of the hepatic lobule undergo different molecular adaptations to metabolic stress. Pericentral hepatocytes undergo lipogenic and xenobiotic pathways, whereas periportal ones show downregulation of mitochondrial β-oxidation genes and activation of inflammatory signaling, thus acquiring a more pericentral-like phenotype. Spatial omics further highlighted the accumulation of triglycerides, ceramides, and reactive lipid species predominantly in the pericentral zone, reflecting localized hypoxia and redox imbalance. This metabolic heterogeneity parallels distinct microenvironments of injury, inflammation, and fibrogenesis, providing mechanistic insight into disease progression. Green arrows represent normal (physiological) expression, while red arrows indicate altered expression either upregulated or downregulated in MASLD. It has been described that portal inflammation is present in 60% of adult MASLD patients, and it is associated with more severe disease and adverse outcomes. Portal inflammation does not correlate with lobular inflammation, thus eluding the NAS scoring system [68]. Accordingly, another study demonstrated that patients without portal fibrosis were protected against cirrhosis complications [69].

These pathways, which feature specific zones of the liver in physiological conditions, may be altered by fat accumulation occurring in MASLD, and changes in the transcriptomic profiles contribute to an uneven lipid distribution, thus guiding the consequent cellular damage and causing HEPs to lose their zonal identity. During MASLD, steatosis originates in the PC zone in the majority of patients, mainly due to the zonal distribution of fatty acid synthesis. The pattern of panlobular steatosis is the second most common, indicating an inter-individual variability in the disease phenotype. Similarly, MASH is initially localized in the PC area, due to the higher oxidative stress and hepatocellular injury in this zone and then spreads throughout the entire lobule. In detail, pericentral hepatocytes activate lipogenic programs, whereas periportal ones downregulate beta-oxidation and start to express typical genes of the pericentral zone, including those involved in de novo lipogenesis. This process is defined as periportal-to-pericentral reprogramming, and it is triggered by hypoxia, oxidative stress, alterations in beta-catenin and PPAR signaling, activation of inflammation and fibrogenesis (Figure 2). The loss of HEPs’ identity and the appearance of hybrid cells with a mixed PC and PP phenotype in response to a maladaptive tissue repair could boost the progression towards advanced stages, such as fibrosis, cirrhosis and HCC, which also foresees the crosstalk between HEPs and NPCs [66]. Indeed, the latter are exposed to the gradient of nutrients and oxygen availability across the lobule. For instance, LSECs physiologically have a greater number of fenestrae in the PC zone since they cooperate in the exchange of metabolites between the blood and hepatocytes. During MASLD progression, LSECs undergo structural changes with the loss of their fenestration and deposition of collagen in the space of Disse, thus creating a hypoxic microenvironment and impacting zonation through HIF-mediated mechanisms [39]. Concerning KCs and immune cells (i.e., T lymphocytes and dendritic cells), they are localized in the PP region, acting as the first defense against gut-derived bacteria byproducts. Their presence in the PP area is further increased in MASLD, together with infiltrating immune cells that also populate the PC zone. Finally, cholangiocytes, which are located in the PP region, contribute to MASLD progression by activating the ductular reaction that leads to severe fibrosis and more so in those patients with a PP involvement [67].

To date, a direct comparison between physiological and MASLD zonation is not completely clarified, especially for NPCs, as a consequence of the difficulty in mapping their zonation profiles due to the abundance of HEPs that mask their heterogeneity. However, single cell technologies paired with spatial omics could allow us to define specific markers of NPCs zonation. For instance, it has been reported that PC LSECs express Wnt-pathway ligands such as *Wnt2*, *Wnt9b*, *Cdh13*, and *Rspo3,* whereas the PP counterpart shows *Dll4*, *Efnb2*, *Jag1*, and *Ltpp4* [70]. During MASH, LSECs increase the expression of *Cxcl9*, a chemokine that recruits macrophages to the liver, thus amplifying inflammation and fibrosis. The *Rspo3* gene is also present in PC HSCs and maintains hepatocyte zonation by modulating Wnt signaling [71]. Conversely, PP HSCs show *Ngfr2*, *Il34* and *Tagln*. Concerning PP KCs, they produce more *TNF-α* and *PGE,* while the PC ones produce more *Il-1,* suggesting a functional zonation of macrophage subsets with more inflammatory properties in the PC region. Notably, when the disease progresses, the disruption of metabolic zonation in both HEPs and NPCs may also be explained by aberrant activation of signaling pathways driving hepatic zonation and cell fate determination, including Wnt/β-catenin, Hh, HIPPO, and Notch, together with the hypoxic microenvironment, and the subversion of hepatic architecture due to fibrotic septa deposition.

Finally, the definition of HEPs and NPCs zonation is crucial for the choice of therapeutic options since it has been described as an asymmetric response to drug action. For instance, FXR, PPARs, and thyroid hormone receptor (TRβ) agonists mainly work in the PC zone, where they are highly expressed in the overfed state. In detail, it has been described that the methylation of binding sites for RXR, which complexes with FXR and PPARs, is strongly zonated. Although RXR is distributed uniformly in the hepatic lobule, its transcription binding sites are predominantly methylated and thus inaccessible in the portal area [72]. Consequently, the MASLD therapeutic options based on FXR agonists and biliary acids (i.e., obeticholic acid) may be more efficacious in the PC zone. Therefore, it is conceivable that other novel RXR agonists (FGF19/21 analogues) might preferentially act in the PC region. Conversely, vitamin E and other antioxidants (i.e., Cysteamine) exert a significant improvement of inflammation in the portal zone where oxidative stress is more pronounced [73].

In conclusion, MASLD is a spatially organized disease characterized by zonal changes in both HEPs and NPCs, in which distinct regions of the hepatic lobule undergo molecular adaptations to metabolic stress. These regional alterations create a heterogeneous microenvironment of steatosis, inflammation and fibrogenesis that might be deeply understood with the application of spatial omics, thus providing novel insights into MASLD pathogenesis.

In addition, the assessment of damage distribution and, even more so, of portal inflammation, which is not currently included in any score, may be useful to foresee the prognosis and to choose a personalized approach targeting the zonal switching. The latter should also be considered in the design of a randomized clinical trial.

## 5. The Advent of Spatial-Omics and Advantages in Hepatology Research

Next-generation sequencing (NGS) techniques have revolutionized basic and clinical research, allowing the determination of the molecular profile of hundreds of thousands of cells, which can be assayed in parallel to understand the intricacies of tissue organization and cell-to-cell communication.

In 2009, Tang and collaborators added another layer of complexity through the advent of single-cell RNA sequencing (scRNA-seq), which overcame the limitations of bulk RNA-seq [74]. While the latter permits extrapolating a large amount of data resulting from an average cell expression profile, the scRNA-seq catches up the transcriptomic information at a single-cell level, thus dissecting cellular heterogeneity and revealing distinct cell types, states, and transitions. For instance, Park et al. demonstrated that HEPs differently responded to nutritional overload by characterizing their transcriptomic landscape through droplet-based scRNA-seq (Drop-seq). They found that hepatocytes undergo extensive transcriptional modifications in response to a high-fat diet (HFD), even if the metabolic changes depended on the hepatic zone considered. Specifically, PC HEPs promote lipid droplets (LDs) formation after HFD challenge, thus upregulating genes of lipid synthesis and ketogenesis. Conversely, PP HEPs are likely resistant to fat accumulation, showing a great increase in gluconeogenesis and down-regulating genes involved in amino acid catabolism and redox status, possibly supporting that this population may be responsible for glucose intolerance in HFD-fed mice [75]. Several scRNA-seq studies have also uncovered the heterogeneity and cell-to-cell interactions of NPCs in MASH mice under an amylin (AMLN) diet. It has also emerged that endothelial cells are zonated in PP and PC areas, as are the hepatocytes. Specifically, PP endothelial cells are involved in hormone signaling and lipid metabolism. Conversely, LSECs, an Endo-subtype, co-localized with HEPs in the PC areas and exert a fundamental role for monocyte recruitment and differentiation in MASH livers through secreting paracrine and autocrine signals [70].

The scRNA-seq innovation was only the first step before researchers realized that higher single-cell resolution techniques would be further required for genomics, proteomics, and metabolomics. The aggregation of these approaches into multi-omics is rapidly growing, attempting to delve deep inside tissue dynamics and cellular interactions, thereby enhancing our understanding of biological networks and disease mechanisms. In this regard, a recent study by Sveinbjornsson et al. integrated genomic, transcriptomic and proteomic analyses to investigate genetic associations related to MASLD and its complications in individuals of European ancestry. The authors utilized large datasets from deCODE Genetics, UK Biobank, and FinnGen cohorts and observed that several polymorphisms (i.e., PNPLA3 I148M, TM6SF2 E167K, rs72613567 in HSD17B13), which are commonly associated with MASLD, further overlapped with other metabolic traits such as BMI, liver enzymes, and diabetes. Furthermore, other missense variants emerged in this study, which correlated with plasma levels of proteins involved in hepatosteatosis, fibrosis, and increased risk of atrial fibrillation and heart failure, thus highlighting how multi-omics may aid in identifying novel biomarkers potentially useful for MASLD diagnosis and treatment [76].

Despite such enormous strides, these technologies are not without their flaws. Among them, isolating single cells for -omic data analysis requires a disruption of the native tissue, thus wasting critical information about tissue architecture, spatial localization of subcellular populations and of their in-situ communications [77]. These limitations have recently been overcome with the development of spatial-omics techniques (genomics, transcriptomics, proteomics, and metabolomics), which allow mapping the molecular profile within tissue structure, preserving spatial relationships and interactions among cells.

Therefore, the purpose of this chapter will be to shed light on the new spatial-omics methodologies, focusing on their advantages in the field of liver disease, particularly in MASLD.

### 5.1. Spatial Transcriptomics: Molecular Fundamentals and Applications in MASLD

In 2016, Ståhl et al. [78] first introduced the visualization and quantitative analysis of the transcriptome with spatial resolution. Specifically, the authors annealed histological sections of mouse brain and human breast cancer to reverse transcription primers linking to unique positional barcodes, thereby revealing an unexpected heterogeneity within the specimens, which would not be possible to detect with traditional RNAseq analysis.

The application of spatial transcriptomics has already yielded significant discoveries in immune modulation and MASLD, thereby uncovering new inflammatory mechanisms such as diverse macrophage populations, self-reactive T cells, the role of unconventional T cells, and interactions between platelets and immune cells. Hendrikx et al. [71] characterized the origin and function of a subclass of monocytes, overexpressing Triggering Receptor Expressed on Myeloid Cells 2 (TREM2) receptor in both MASH rodents and humans. Through spatial transcriptomics, they revealed that TREM2^+^ macrophages derive from bone marrow and localize near the areas of hepatocellular damage, inflammation, and fibrosis. Mechanistically, the authors further demonstrated that *Trem2*-deficient mice enhanced T-cell infiltration within the hepatic tissue, thereby supporting that TREM2+ macrophages may exert a protective effect against inflammatory response [70,71,79,80]. Accordingly, serum measurement of TREM2 in two independent cohorts, including biopsy-proven MASLD patients and subjects with non-invasive MASLD diagnosis, resulted in a good biomarker for MASLD advanced stages, potentially becoming a candidate target for therapeutic interventions [71].

In eight liver explants from cirrhotic patients, Chung and collaborators combined spatial transcriptomics with scRNAseq (referred to as deconvolution analysis) to accurately assign gene expression data to specific cell types and locations within the liver. ACTA2+, FABP4+, and COL3A1+ mesenchymal cells, IL17RA+ S100A8+ and FCER1G+ tissue monocytes, VCAM1+ SDC3+ KCs, CCL4+ CCL5+ KLRB1+, and GZMA+ IL17RA+ T cells and HLA-DR+, CD37+ CXCR4+, and IGHM+ IGHG+ B cells were markedly represented in fibrotic areas compared to nonparenchymal regions of cirrhotic explants. The significant enrichment of NPC subpopulations found in this study, on one side, delineates a precise map of cellular and molecular heterogeneity within fibrotic liver specimens and, on the other side, opens new therapeutic windows [81].

In addition, spatial transcriptomics revealed that glycolysis in HSCs amplifies liver fibrosis by promoting fibrogenic extracellular vesicle release in the hepatic PC zone, which represents a potential therapeutic target [82].

Liu et al. [83] further investigated the role of different cellular components resident in the tumor microenvironment (TME) on immunotherapy response in HCC patients receiving the anti-PD-1 treatment, an immune checkpoint inhibitor. Spatial niches were found in TME, enriched with SPP1+ macrophages and cancer-associated fibroblasts (CAFs). SPP1+ macrophages stimulate CAFs to remodel the extracellular matrix and to produce a tumor immune barrier (TIB), an immunosuppressive structure. The latter dampens the interaction among CD8+ T cytotoxic and malignant cells in the tumor core and limits the anti-PD-1 efficacy. Consistently, anti-SPP1 treatment enhanced anti-PD-1 efficacy by troubling SPP1+ macrophages-CAFs interaction, TIB formation and promoting CD8+ T infiltration in cancerous tissue, thereby highlighting future directions for therapeutic strategies which disrupt cell-cell communication in HCC [83].

### 5.2. Spatial Proteomics

As spatial transcriptomics detects a proxy for molecular function, the location of the transcripts is not as functionally informative as that of proteins [84]. Although transcriptome and proteome comparative analyses have a high degree of concordance, up to 10% of transcribed genes are regulated at the post-transcriptional level [85]. Human eukaryotic cells express an average of 10,000 proteins or more, whose functions are tightly linked to their subcellular localization within membrane-bound organelles (such as endoplasmic reticulum, Golgi apparatus, and mitochondria), or non-membranous compartments (i.e., centrosomes). The proteins’ distribution is dynamic, changing within individual cells, during cell-cell interactions or in response to endogenous/exogenous stimulations. Inverso et al. [85] combined spatial cell sorting with transcriptomics and proteomics/phosphoproteomics to understand protein regulation in the liver vasculature. They found that the phosphorylation of receptor tyrosine kinases was detected preferentially in the PC area and identified Tie receptor signaling as a specific regulator of vascular Wnt activity, which orchestrates angiocrine signaling, thereby controlling hepatocyte function during liver regeneration. 

Weiss and colleagues introduced an advanced single-cell Deep Visual Proteomics (scDVP) workflow, which integrates high-resolution microscopy with artificial intelligence (AI)-guided image analysis, automated laser microdissection and ultra-sensitive mass spectrometry (MS) at single cell resolution, enabling spatially resolved proteomic analysis of HEPs in human liver tissue. They found that half of the hepatic proteome is expressed with spatial variation, and 171 proteins were found to be associated with a strong zonation profile with distinct metabolic functions assigned to PP and PC regions. Moreover, they demonstrated that fibrosis disrupts lobular architecture, obstructing blood flow, with consequent changes in the oxygen gradient, particularly affecting PC proteins and pathways such as xenobiotic and retinol metabolism [86].

In the perspective that a multi-omics approach may be more informative, Guilliams and colleagues exploited spatial proteogenomics, which combined spatial transcriptomics and proteomics to generate a cellular atlas of human liver and unravel the cell-cell circuits. They identified lipid-associated macrophages (LAMs) around the bile ducts, which are preferentially recruited into the steatotic regions of the liver and are activated by lipid exposure. Moreover, they described that KCs activation requires their interaction with HSCs via the ALK1-BMP9/10 axis [87].

Another application of spatial proteomics is represented by its combination with imaging techniques and functional assays for the study of organelles’ heterogeneity according to their spatial context. In this regard, Kang et al. [88] compared mitochondrial composition patterns and the post-translational alterations of structural proteins of mitochondria across the hepatic lobule. These authors determined that mitochondrial morphology changes across the PP–PC axis, showing larger spherical organelles in PP HEPs, whereas longer tubulated organelles in PC ones. The former morphology was associated with enhanced OXPHOS capacity and bioenergetic efficiency, whereas the second protects against mitochondrial degradation and correlates with higher enzymatic activities. This mitochondrial heterogeneity aligns with metabolic zonation. Indeed, it has been reported that mitochondrial DNA copies are more abundant in midlobular and periportal regions, thus paralleling the porto-central oxygen gradient [72].

### 5.3. Spatial Metabolomics and Lipidomics

In recent years, other forefront omics, including spatial metabolomics and lipidomics, have contributed to giving more insights into the pathophysiology of MASLD. Seubnooch et al. exploited MS imaging to map lipid species across the lobule by discriminating 130 lipids with zone-specific signatures in MASH [89]. In addition, the same authors found an enrichment of fatty acids in the PP region, whereas phospholipids were also distributed in the PC zone. Notably, the PI(36:2), PI(36:3), PI(36:4), PI(38:5), and PI(40:6) phosphatidylinositols were located predominantly in the midzone and triacylglycerols/diacylglycerols in the PC area where de novo *lipogenesis* occurs [90]. However, during MASH, triacylglycerols/diacylglycerols shift towards the PP zone together with saturated and monounsaturated fatty acids [89]. Furthermore, changes in zonation of phospholipids have been related to membrane remodeling and eicosanoid formation. In particular, it has been reported that during MASH, the lipid zonation is lost and the arachidonic acid release from PC hepatocytes is exacerbated through LPCAT2, thus favoring pro-inflammatory eicosanoid production and oxidative damage in the PC zone [91]. These events may possibly explain the recruitment of macrophages and their M1 polarization in PC steatotic regions during the switching to MASH.

Accordingly, alterations in phospholipid zonation have also been reported by Wattacheril et al. [92] who postulated that their specific abundance and distribution may be associated with the histological grade of the disease. In keeping with this notion, the zonal distribution of various phosphatidylcholines and of enzymes governing their biosynthetic pathways is lost in MASH specimens.

Notwithstanding, the combination of spatial metabolomics, lipidomics and transcriptomics has been recently applied by Otkur and colleagues to point out the disruption of zonation in a GRP35-dependent manner [93]. These authors described for the first time the role of this protein, which connects lipid metabolism and inflammation, by influencing sphingolipid metabolites like ceramides and sphingomyelin, thereby affecting liver zonation. Accordingly, even Ochoa-Rios et al. reported altered glycan profiles in mice and humans affected by MASH and linked them to fibrosis severity and to the evolution of liver damage up to cancer [94].

Intriguingly, spatial lipidomics found its utility also in the study of LDs formation, in the definition of their dimension and in the study of enzymes involved in their remodeling (i.e., LPCAT2). In this regard, previous studies reported the composition of giant LDs as enriched in saturated triglycerides [95]. The latter are frequently observed in patients with MASH and severe fibrosis, thus possibly linking enlarged LDs, saturated lipids and disease progression. Conversely, lipid loading in samples with mild steatosis is primarily detected in the PC zone in line with its higher expression of genes related to de novo *lipogenesis* in this region. To further dissect the impact of metabolic zonation in the tissue microenvironment, Yuang and colleagues employed the spatial single nuclear metabolomics (SEAM), which is a method that couples MS and computational algorithms to enrich higher resolution [96]. This forefront technique allows the identification of subpopulations of HEPs with a peculiar metabolic landmark, localized near fibrotic niches in human livers.

### 5.4. Other Spatial Approaches (Spatial Genomics/Epigenomics)

By pairing spatial transcriptomics with epigenetic data, it is possible to investigate chromatin accessibility and epigenetic marks and correlate them with transcriptomic perturbations directly within the structural context. In this regard, Brosch and collaborators identified a porto-central gradient of DNA methylation at binding sites of different transcription factors, including those regulating Wnt signaling activation [72]. The application of these technologies has acquired more value in the field of cancer, where it enabled the characterization of distinct cancerous clones and their copy number variations (CNVs) or clone-specific genetic alterations in the local tumor microenvironment [97]. Here, spatial ATAC has been applied to study chromatin remodeling during the course of organogenesis and tumor development [98].

Spatial approaches have also been employed to investigate noncoding RNAs and viral RNAs to study tissue regeneration and local response to different viral abundance [99].

These techniques have been poorly explored in the context of hepatic zonation. However, it is conceivable that spatial investigations of chromatin modifications may be helpful in better understanding the subversion of metabolic pathways across the different zones of the lobule.

## 6. Conclusions

Although liver zonation was described a century ago, the application of omics-technologies permits us to deeply understand how liver anatomy and the gradient of oxygen, nutrients and hormones affect HEPs and NPCs functions. The characterization of the spatial arrangement of the hepatic lobule and of the cell-cell interactions is crucial to figure out the pathogenesis of MASLD and its evolutive forms. In particular, it allows the identification of the specific metabolic/transcriptomic/proteomic/lipidomic reprogramming that cells undergo in a zone-dependent manner during disease progression. MASLD pathogenesis is complex and entails several cues, including genetic, epigenetic, and environmental risk factors. Thus, the integration of multiple omics approaches, including spatial proteogenomics, metabolomics and lipidomics, coupled with recently emerged AI-based data analysis, will boost the discovery of novel zone-specific biomarkers and pharmacological approaches targeting preferential areas of the hepatic lobule. Furthermore, outlining the spatial resolution of the disease will provide more informative clinical records to be employed in the diagnosis and in the design of clinical trials. In this regard, the assessment of portal vs pericentral inflammation, which is not currently included in any score, may be useful to foresee the disease outcomes and to identify novel personalized strategies aimed at targeting the zonal switching.

## Figures and Tables

**Table 1:** Marker genes of liver zonation and biological processes.

		Zone	
	Pericentral	Midlobular	Periportal
**Marker Genes**	*AHR*, *ALDH3A2*, *CSAD*, *CYP1A2*, *CYP2A5*, *CYP2C*, *CYP2E1*, *CYP3A*, *CYP3A4*, *CYP4A14*, *GCK*, *GLUL*, *GS*, *GSTM1*, *LDHD*, *LECT2*, *MUP17*, *OAT*, *SLC1A2*, *IGFBP1*, *NT5E*, *ADHA4*, *BCHE*	*HAMP*, *HAMP2*, *IGFBP2*, *CYP8B1*, *HINT1*, *COX7C*, *APOC1*, *FABP1*, *MT2A*, *MT1G*, *NDUFB1*	*CPS1*, *SDS*, *CYP2F2*, *HAL*, *GLS2*, *ASS1*, *HSD17B13*, *HSD17B6*, *ALDH1B1*, *ARG1*, *PCK1*, *SULT5A1*, *MFSD2A*, *BTNL9*, *ANPEP*, *MTHFS*, *GATM*, *L-FABP*, *SOX9*, *E-CDH1*, *MUP1*, *GSTP1*, *HPX*, *ALDOB*, *FBP1*, *MUP21*, *IGF1*, *CDH1*, *CYP7A1*, *HSB3B7*, *HMGCS1*
**Biological Process**	Bile acid synthesis Glutamine synthetase Glycolysis Lipogenesis Xenobiotic biotransformation reactions Ketogenesis		Cholesterol synthesis Fatty acid oxidation Gluconeogesis Urea and protein synthesis Glutaminolysis

## Data Availability

No new data have been created.

## Abbreviations

The following abbreviations are used in this manuscript:

MASLD	Metabolic dysfunction-associated steatotic liver disease
MASH	Metabolic dysfunction-associated steatohepatitis
SLD	Steatotic liver disease
HCC	Hepatocellular carcinoma
IR	Insulin resistance
T2DM	Type 2 diabetes mellitus
ROS	Reactive oxygen species
FFA	Free fatty acids
mtDNA	Mitochondrial DNA
ER	Endoplasmic reticulum
LSECs	Liver Sinusoidal Endothelial cells
HSCs	Hepatic stellate cells
KCs	Kupffer cells
PC	Pericentral
PP	Periportal
Hh	Hedgehog
GSK3	Glycogen synthase kinase-3
APC	Adenoma polyposis coli
DVL	Dishevelled
LRP5/6	LDL receptor–related protein 5/6
KO	Knockout
Rspo	Rspondin
TCF	T-cell factor
NGS	Next-generation sequencing
scRNA-seq	Single-cell RNA sequencing
Drop-seq	Droplet-based scRNA-seq
HFD	High-fat diet
LDs	Lipid droplets
AMLN	Amylin
TREM2	Triggering Receptor Expressed on Myeloid Cells 2
TME	Tumor microenvironment
CAF	Cancer-associated fibroblasts
TIB	Tumor immune barrier
scDVP	Single-cell Deep Visual Proteomics
AI	Artificial intelligence
MS	Mass spectrometry
LAMs	Lipid-associated macrophages
LPCAT2	Lysophosphatidylcholine Acyltransferase 2
GRP35	G-Protein-Coupled Receptor 35
SEAM	Spatial single nuclear metabolomics
CNVs	Copy number variations
